# Linear and Circular UWB Millimeter-Wave and Terahertz Monostatic Near-Field Synthetic Aperture Imaging

**DOI:** 10.3390/s20061544

**Published:** 2020-03-11

**Authors:** Jose Antonio Solano-Perez, María-Teresa Martínez-Inglés, Jose-Maria Molina-Garcia-Pardo, Jordi Romeu, Lluis Jofre, José-Víctor Rodríguez, Antonio Mateo-Aroca

**Affiliations:** 1 Departamento Tecnologías de la Información y las Comunicaciones, Universidad Politécnica de Cartagena, Cartagena, 30202 Murcia, Spain; josemaria.molina@upct.es (J.-M.M.-G.-P.); jvictor.rodriguez@upct.es (J.-V.R.); 2 Centro Universitario de la Defensa, Universidad Politécnica de Cartagena, Base Aérea de San Javier. Academia General del Aire, 30720 Murcia, Spain; mteresa.martinez@cud.upct.es; 3 CommSenslab, Department of Signal Theory and Communications, School of Telecommunications Engineering Technical University of Catalonia (Universitat Politecnica de Catalunya, UPC) Campus Nord UPC, Edif. D-3 Jordi Girona, 1-3, 08034 Barcelona, Spain; romeu@tsc.upc.edu (J.R.); jofre@tsc.upc.edu (L.J.); 4 Departamento Tecnología Electrónica, Universidad Politécnica de Cartagena, 30202 Cartagena, Murcia, Spain; Antonio.Mateo@upct.es

**Keywords:** imaging, millimetric wave, terahertz, measurements

## Abstract

Millimeter-wave and terahertz frequencies offer unique characteristics to simultaneously obtain good spatial resolution and penetrability. In this paper, a robust near-field monostatic focusing technique is presented and successfully applied for the internal imaging of different penetrable geometries. These geometries and environments are related to the growing need to furnish new vehicles with radar-sensing devices that can visualize their surroundings in a clear and robust way. Sub-millimeter-wave radar sensing offers enhanced capabilities in providing information with a high level of accuracy and quality, even under adverse weather conditions. The aim of this paper was to research the capability of this radar system for imaging purposes from an analytical and experimental point of view. Two sets of measurements, using reference targets, were performed in the W band at 100 GHz (75 to 110 GHz) and terahertz band at 300 GHz (220 to 330 GHz). The results show spatial resolutions of millimeters in both the range (longitudinal) and the cross-range (transversal) dimensions for the two different imaging geometries in terms of the location of the transmitter and receiver (frontal or lateral views). The imaging quality in terms of spatial accuracy and target material parameter was investigated and optimized.

## 1. Introduction

The progressive shift toward millimetric and terahertz frequencies is opening up new possibilities for the use of electromagnetic waves to optically explore opaque metallic or non-metallic objects and recover their shape, as well as provide the characteristics of penetrable non-metallic objects. The applications cover two main areas. The first is as a high-resolution synthetic aperture radar with imaging capabilities for use as a new sensing device in the next generation of vehicles [1]. The second area is related to imaging applications for object characterization, such as the non-destructive visualization of structures, the composition of chemical compounds, the identification of dangerous products, and the identification of hidden objects or pathological parameters in biological tissues. Although the resolution can be exceeded by optically based systems, the terahertz frequency provides resolutions on the order of millimeters, which is enough for many applications, with the advantage of a better penetrability in certain cases [2].

Considering the applications identified previously, the contribution of the following works that provided background about the state of the art is relevant. In Reference [3], a synthetic aperture radar (SAR) was used for an imaging system using a specific algorithm, demonstrating the capability of recovering scattering information from a system operating at 36.5 GHz. In Reference [4], a terahertz inspection system for radome polymer object inspection operating from 70 to 110 GHz and from 110 to 170 GHz was presented with very good resolution results, proposing the use of higher frequencies around 300 GHz for better resolution. In Reference [5], a terahertz three-dimensional (3D) linear frequency modulated (LFM) SAR imaging system for security detection at the 340 GHz band was tested using a two-dimensional (2D) Fourier transform. In Reference [6], a radar operating at 522 GHz was presented in order to explore this band. Finally, in Reference [7], a new reconstruction technique operating in the time domain and in the microwave and millimeter wave band was presented.

In this context, imaging radars are capable of obtaining the spatial distribution of the reflectivity of an object. This technique enables the material characterization and non-destructive evaluation of objects. Additionally, this radar system can also capture the image of an object that may be optically blocked by one or more barriers, such as in radars penetrating the ground, the visualization of hidden objects, and vision through walls. Millimeter-wave (mmW) systems, that is, systems operating in the W band and terahertz frequency band, combine the penetrability potential and the required resolution to accomplish the exploration of opaque metallic or non-metallic objects, as well as recover the shape and characteristics of non-metallic objects. The measurements were performed using a vector network analyzer (VNA), connecting each port to specific antennae operating as the transmitter (*Tx*) and the receiver (*Rx*), with the aim of obtaining the scattering parameters, working in the frequency domain [8].

Most of the current mmW automotive radar systems work in the W band (70–110 GHz), while future systems may extend to the upper part of the extremely-high-frequency (EHF) (30–300 GHz) region. In order to explore the capabilities of operative radar systems at those high frequencies, it becomes necessary to study the scattering and imaging performance of some of the basic geometries found in vehicular scenarios. In terms of scattering, when moving toward these higher frequencies, such as 300 GHz, the smooth surface requirements of λ/32 may represent 30 microns, and sensitivity to low-contrast material is a key item to test for imaging systems. Subsequently, material rugosity and low-permittivity materials should be tested in order to approach realistic conditions in the experiments. 

This paper presents the principle of operation of a mmW imaging radar system that allows the analytical and experimental assessment of very flexible geometries adaptable to different possible realistic situations, as well as an image reconstruction from the frontal and lateral views of moving vehicles using low-permittivity targets. Then, the use of a double focal system is proposed that is independent of the transmitter and receiver. This scanning system can be used in a wide variety of geometries applying near-field techniques with synthetic aperture processing. 

The aim of this work is to perform a detailed review of the use of a monostatic geometry as opposed to previous systems, such as those based on the approximation of multiple plane waves [8]. To achieve this goal, a single-frequency system developed previously [9] is extended to the mmW and terahertz frequency band to exploit the benefits of these new frequencies.

This paper is organized as follows: Section 2 explains the theoretical formulation related to imaging techniques using a near-field approach and synthetic aperture. Section 3 presents the experimental results for the test cases. Section 4 discusses the results. Section 5 states the conclusions and summarizes the contribution.

## 2. Materials and Methods

### 2.1. Formulation

**Figure 1:**
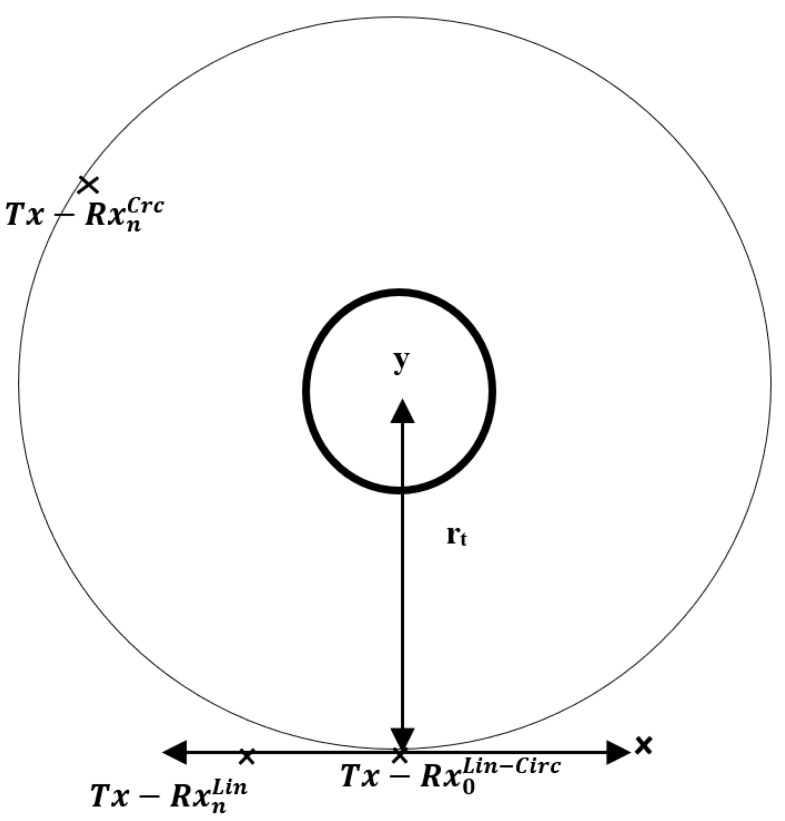
Scheme of measurement geometries. Circular geometry of radius $r_{t}$ being $Tx-\;Rx{\;}_{n}^{Crc}\;antenna\;positions\;marked\;as\;“x”$ and linear geometry being $Tx-\;Rx{\;}_{n}^{Lin}\;antenna\;positions\;marked\;as\;“x”$ at a distance $r_{t}$ to the target, being $Tx-\;Rx{\;}_{0}^{Lin-Circ},$ i.e., the coordinate origin.

The near-field imaging system presented here is based on a flat synthetic aperture radar system that combines a *Tx* (transmitter) antenna and an *Rx* (receiver) antenna placed close together. There are two different arrangements for the movement of the *Tx*–*Rx* set around the object, as shown in Figure 1. The measurement geometry is based on the *Tx* antenna and the *Rx* antenna maintaining a fixed relative position relative to the other during the exploration process. This is a monostatic measurement with a certain bi-static angle. In the linear geometry, the *Tx*–*Rx* set explores in the vertical plane, i.e., the *zx* plane in Figure 1, while, in the circular geometry, the *Tx*–*Rx* set is moved around the object at a constant distance r_t_. 

The goal is to obtain an image of the spatial distribution of the contrast of the object being tested, defined as $c\left(\vec{r}\right)=\left(\varepsilon_{t}\left(\vec{r}\right)-\varepsilon_{b}\right)/\varepsilon_{b}$, where $\varepsilon_{t}\left(\vec{r}\right)$ is the dielectric permittivity of the object and $\varepsilon_{t}\left(\vec{r}\right)$ corresponds to the background.

Let us consider an incident or illuminating field polarized along the *y*-axis (vertical axis) on a cylindrical object that is invariant along the vertical axis. In this way, the problem can be treated as a scalar problem. We denote the field produced by a transmitting element located at r →_(t_i) at a frequency f in the field point ${\vec{r}}_{t_{i}}$ as $\vec{E}\left(\vec{r},f;{\vec{r}}_{t_{i}}\right)$. According to the principle of equivalence [5], the illuminating field induces in the object a current distribution proportional to the electric contrast, which can be expressed as

(1) $${\vec{J}}_{eq}\left(\vec{r},f;{\vec{r}}_{t_{i}}\right)=j\omega \varepsilon_{b}c\left(\vec{r}\right)\vec{E}\left(\vec{r},f;{\vec{r}}_{t_{i}}\right)$$

This equivalent current can be understood as the source of the scattered field produced by the object and as a “trace” or image of the original object.

As shown in Figure 1, a *Tx* antenna, an *Rx* antenna, and the frequency converters are moved using a circular geometry around a circle of radius $r_{t}$, or using a linear geometry, along a set of positions $\left(N_{x},N_{z}\right)$ along the x- and z-axes, respectively, by collecting a two-dimensional array of monostatic measurements. Linear geometry is one approach of the configurations used for a radar system placed in a vehicle.

The scattered field generated by the equivalent current ${\vec{J}}_{eq}\left({\vec{r}}_{t},f;{\vec{r}}_{t_{i}}\right)$ at each point ${\vec{r}}_{t_{i}}$. of the target and measured in the *Rx* antenna at each position is written as

(2) $${\vec{E}}_{s}\left({\vec{r}}_{R_{j}},f;{\vec{r}}_{t_{i}}\right)=-j\omega \mu_{0}\underset{V_{0}}{\int }{\vec{J}}_{eq}\left({\vec{r}}_{t},f;{\vec{r}}_{t_{i}}\right)G\left(\left|{\vec{r}}_{R_{i}}-{\vec{r}}_{t}\right|,\;f\right)dV$$

where $G\left(\left|{\vec{r}}_{R_{i}}-{\vec{r}}_{t}\right|,f\right)$ is Green’s function for the corresponding geometry. In the case of a 3D geometry, G(r)=e−jkbrr, where $k_{b}=\omega \sqrt{\mu_{0}\varepsilon_{0}\varepsilon_{b}}$. Under the Born hypothesis or a point-like distribution of scatterers, the illuminating field within the target is approximated by the incident field. Using low-directivity antennas that uniformly illuminate the target, the scattered field measured by the *Rx* antenna at each position is found as

(3) $${\vec{E}}_{s}\left({\vec{r}}_{R_{j}},f;{\vec{r}}_{t_{i}}\right)=-k_{b}^{2}\left(f\right)A_{d}\underset{V_{0}}{\int }c\;\left(\vec{r},f\right)G\left(\left|{\vec{r}}_{t}-{\vec{r}}_{t_{i}}\right|,f\right)\;G\left(\left|{\vec{r}}_{R_{i}}-{\vec{r}}_{t}\right|,f\right)dV$$

where $A_{d}$ is a complex constant that considers the different constant parameters of the *Tx* antenna and the *Rx* antenna.

The reconstruction process is based on an extension of the multi-frequency case of the bi-focusing technique presented in References [10,11,12]. The contrast factor $c\left(\vec{r}\right)$, averaged over the mmW and terahertz frequency bands and over the whole reconstruction space, is written as

(4) $$c\left(\vec{r}\right)=A_{i}\sum_{f_{min}}^{f_{max}}\sum_{i=1}^{N_{T-R}}\frac{{\vec{E}}_{s}\left({\vec{r}}_{R_{j}},f;{\vec{r}}_{t_{i}}\right)}{k_{b}^{2}\left(f\right)}e^{jk_{b}\left(f\right)\left(\left|{\vec{r}}_{t}-{\vec{r}}_{t_{i}}\right|+\left|{\vec{r}}_{R_{i}}-{\vec{r}}_{t}\right|\right)}$$

where $A_{i}$ is a complex constant that considers all factors of the system’s *Tx–Rx* chain. The use of fast Fourier transform (FFT) to perform the summations in Equation (4) enables the calculation times to be reduced.

### 2.2. Measurement Equipment

The measurements were performed using a commercial network analyzer model ZVA 67 manufactured by Rohde Schwarz. Additionally, the VNA ports were connected to the *Tx* antenna and *Rx* antenna through frequency converters operating from 75 to 110 GHz and from 220 to 330 GHz [13] to allow us to extend the frequency band. The set-up was based on that developed in Reference [14], where a bi-static radar was implemented, using one of the heads to transmit and one of the heads to receive, and measuring the scattering parameter or S_21_. Each head was composed of one antenna and one frequency converter.

### 2.3. Measurement Scheme

The aim of this work is to evaluate the capabilities of a millimeter-wave near-field synthetic aperture imaging radar to accurately reconstruct reference targets under the circular and linear scenarios presented in Figure 1. 

The linear measurement geometry simulates the point of view of an ultrawide-band (UWB) millimeter-wave and terahertz monostatic near-field synthetic aperture imaging system installed in a vehicle in movement. The linear geometry matches the lateral view of the vehicle when moving parallel to a sidewalk or parking area. The object is placed atop a tripod located 1 m from the *Tx* antenna and *Rx* antenna. The *Tx* antenna and its frequency converter are separated from the *Rx* antenna and its frequency converter by 0.112 m, and both elements are mounted on a tray that moves together using a linear positioner [15].

The circular measurement geometry simulates when the vehicle partially turns around an object, such as at a corner or a roundabout. It is approached with a partial rotation of 340°.

### 2.4. Measurement Configuration

The measurements were performed by sweeping the frequency band from 75 GHz to 110 GHz and from 220 GHz to 330 GHz with the two different heads as frequency converters. The models were an R&S (Germany)^®^ZVA-Z110E Converter, W-Band WR-10, and an R&S (Germany)^®^ZVA-Z325 Converter, J-Band WR-03 [13].

Table 1 and Table 2 summarize the vector network analyzer configuration values for 100 GHz and 300 GHz, respectively.

For 100 GHz, commercial horn antennas (model 27240-20, manufactured by Flann Microwave LTD [16]) were used as the *Tx* and *Rx* antennae (one for each purpose). Their frequency range is from 73.8 GHz to 112 GHz, with a gain and E and H plane beamwidth of 18.12 dBi, 22.9°, and 22.3°, respectively, at 73.8 GHz, and 21.41 dBi, 15.1°, and 14.7° at 112 GHz.

For 300 GHz, the 32240-25 model was used, with a frequency range between 217 GHz and 330 GHz, with a gain and E and H plane beamwidth of 23.70 dBi, 11.9°, and 11.9°, respectively, at 217 GHz, and 26.99 dBi, 7.8°, and 7.8° at 330 GHz.

The measurement time, sweeping 8192 frequencies, was 5 s per scanning point. The separation between each frequency complied with the sampling criteria defined in Reference [12].

### 2.5. Measured Objects

The linear measurement geometry was tested using four objects: a “post-it” pack (made of paper), a green cube (non-solid and made of plastic), an empty cardboard box (made of paperboard), and the same cardboard box with a plastic ball inside. The objects were made of non-metallic materials to allow us to check the performance in terms of the sensitivity of this imaging system under the worst conditions at different frequencies. Then, objects made of low-contrast material were selected, such as wax candles $\varepsilon_{r}^{\prime }=2.5$, paper $\varepsilon_{r}^{\prime }=3.7$, and a plastic ball $\varepsilon_{r}^{\prime }=2.2\;\mathrm{to}\;2.6$ [17]. Table 3 identifies the respective objects at both frequencies, the distance d in the *x*-axis used to explore the object at both frequencies, the number of observed positions in the *x*-axis, and the object size.

**Figure 2:**
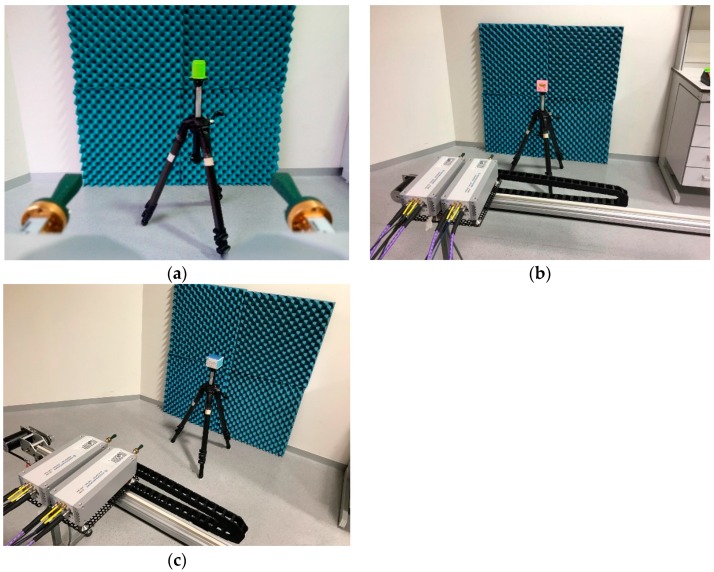
Linear measurement arrangements: (**a**) green cube; (**b**) post-it; (**c**) box.

Figure 2 shows the arrangement for the linear measurement of the three objects.

**Figure 3:**
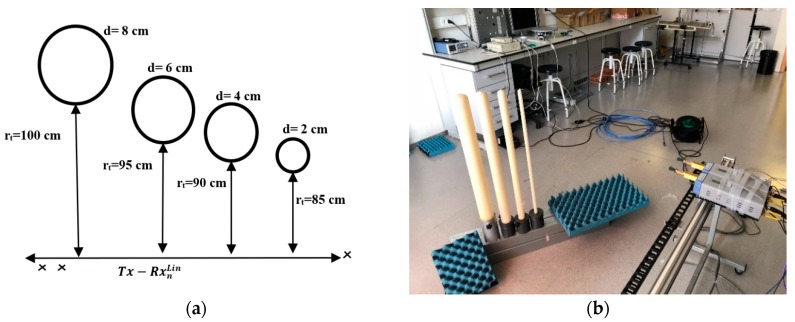
Linear measurement arrangement for the four candles: (**a**) theoretical scheme; (**b**) laboratory photo during measurements.

Additionally, to complete the linear measurements, cylindrical wax candles of different sizes were used as targets. Wax candles with $\varepsilon_{r}=\;2.5,\;tg\delta =0.02$ [17] were placed 100 cm from the measuring array, as depicted in Figure 3. The number of scanning points was 940 points, with a separation between each scanning point of 1 mm.

**Figure 4:**
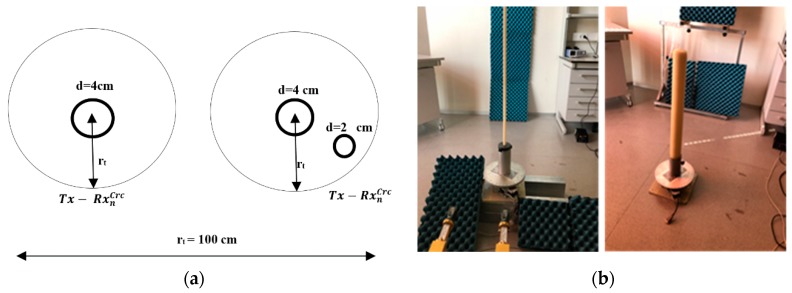
Circular measurement arrangement using candles: (**a**) theoretical scheme; (**b**) laboratory photo during measurements.

The circular measurement geometry was tested using candles as targets due to their geometry. Figure 4a depicts the two different schemes used for measuring. Figure 4b shows an image of the two candles installed in the supporting systems. The number of scanning points was 340 points with a separation of 1°. Then, there was a partial rotation with the aim of checking this lack of information about the object and the final performance.

## 3. Results

**Figure 5:**
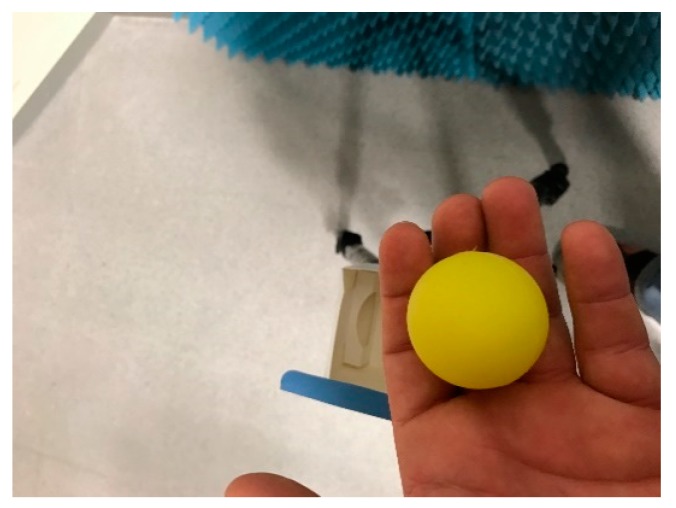
The yellow ball placed in the box.

**Figure 6:**
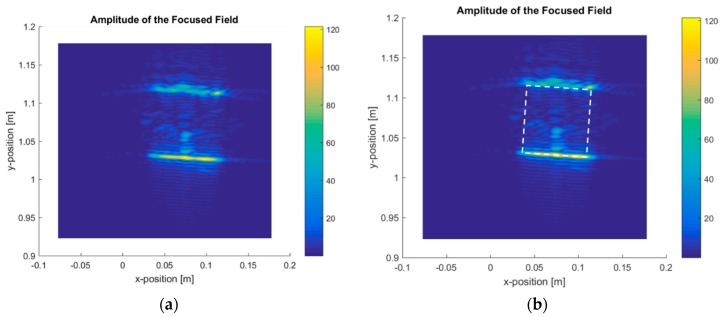
Focused power map of the ball inside a box at 100 GHz: (**a**) raw image; (**b**) raw image overlaid with the box to show accuracy.

**Figure 7:**
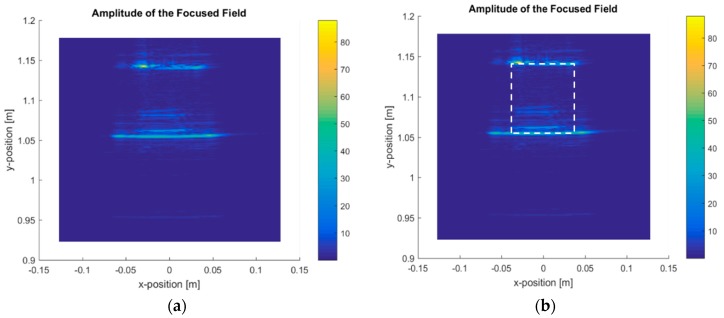
Focused power map of the ball inside a box at 300 GHz: (**a**) raw image; (**b**) raw image overlaid with the box to show accuracy.

**Figure 8:**
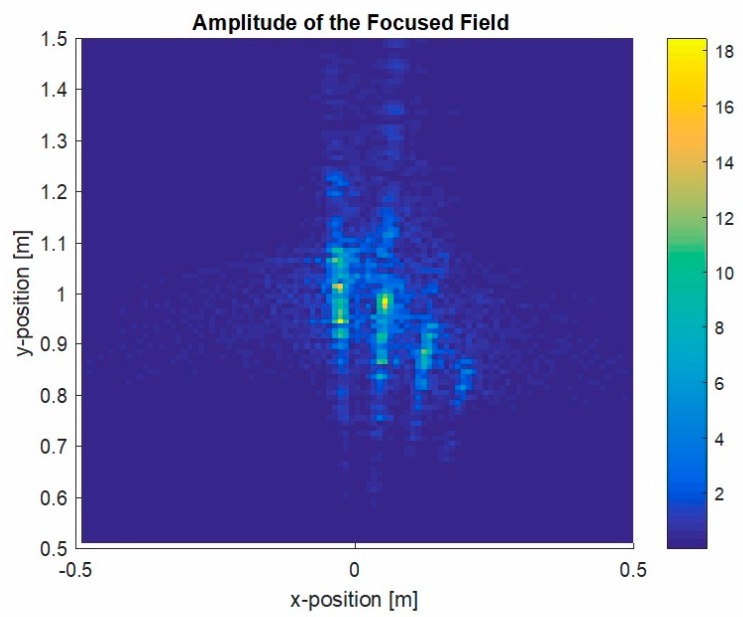
Focused power map of the four candles at 100 GHz using linear geometry.

**Figure 9:**
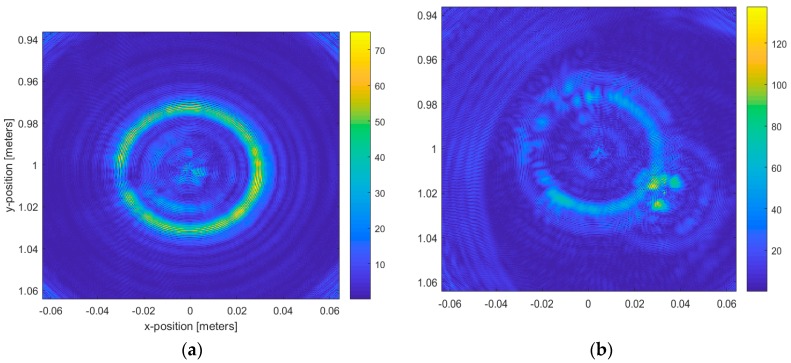
Focused power map of candles at 100 GHz using the circular geometry: (**a**) image reconstruction for a 4-cm candle; (**b**) image reconstruction for two candles: one with a 4-cm diameter and the other with a 2-cm diameter.

Related to the tested objects and the results to be presented, it is clarified that each object was selected to be measured in one specific measurement geometry due to its specific characteristics. The rationale for assigning each object to a specific measurement geometry was the following:

- Linear measurement using cardboard box with a ball, green cube, and post-it. The most representative result was the cardboard with a ball inside. The main idea of the linear measurement of the box with a ball inside was to confirm that this imaging system is capable of detecting and displaying the box, as well as the presence of something inside (Figure 5, Figure 6 and Figure 7). The other two objects were measured but they were considered not so relevant to include in this section.
- Linear measurement of four wax candles (Figure 8). Wax candles are low-permittivity objects, and the sensitivity of the imaging system was tested. The linear measurement is one of the potential geometries when the imaging system is installed in a vehicle.
- Circular measurement of wax candles (Figure 9). The reason for testing the circular measurement of candles is to test the approach of gathering information around the object to be reconstructed using the imaging algorithm.

For the linear measurement geometry, the obtained results are presented for the box with a ball inside (Figure 5) and the four candles (Figure 8).

Figure 6 shows the box-focused power map for 100 GHz, and Figure 7 shows the same for 300 GHz, with the raw image and the image identifying the box.

Figure 9 displays the reconstructed image for the circular geometry using a single candle with a diameter of 4 cm and another measurement of two candles close together.

## 4. Discussion

For the imaging geometries, real vehicular movements may be linear (straight streets) or circular (i.e., intersections or roundabouts). In order to approach realistic conditions, a set of experimental measurements was performed with low-permittivity targets (wax $\varepsilon_{r}^{\prime }=2.5$, paper $\varepsilon_{r}^{\prime }=3.7$, plastic ball $\varepsilon_{r}^{\prime }=2.2\;\mathrm{to}\;2.6,$ surface roughness ~100 μm) for the two basic linear and circular geometries. The sensitivity of the imaging system to low-contrast materials was tested in these measurements. The objects were non-metallic low-relative-permittivity-environment objects, whereby the permittivity was in the 2–4 range. In order to obtain a good resolution and good performance with low-permittivity objects, an absolute bandwidth of 35 GHz was utilized, corresponding to a fractional bandwidth of 38% at 100 GHz. At 300 GHz, the absolute bandwidth was 110 GHz with a fractional bandwidth of 40%. These figures of absolute and fractional bandwidth confirm the ultrawide-band characteristic of the tested imaging system [12].

Figure 6 and Figure 7 show that the imaging algorithm accurately reconstructs the box faces parallel to the *x*-dimension. It is not possible to obtain images from the box faces parallel to the *y*-axis as this area was not illuminated by the measurement heads. The box dimensions were measured, as shown in Figure 6 and Figure 7, and the values matched the actual dimensions of the box. As expected, the measurement conducted at 300 GHz system showed better performance in terms of resolution, providing a more accurate image, as seen in Figure 7. The improved performance for the transverse resolution, along the *x*-axis, of the system operating at 300 GHz versus the system operating at 100 GHz was due to the shorter wavelength in the upper frequency, but the most important difference was the bandwidth, defined as the difference between the final frequency and the initial frequency for each case. In the 100 GHz measurements, the bandwidth was 35 GHz, meaning that the radar swept 35 GHz to obtain the scattered fields of the object. Meanwhile, in the 300 GHz measurements, the bandwidth was 110 GHz, improving the resolution. 

Concerning the identification of the ball inside the box, the reconstructed image (see Figure 6) showed spots/lines inside the box for the 100 GHz and 300 GHz systems. These spots represent the front view of the ball. The ball geometry scattered the energy in all directions, and the measurement system only captured the energy that returned to the bi-static radar. It was not possible to reconstruct the circular shape using a linear measurement geometry.

Continuing with the linear measurement geometry, Figure 8 shows the image of the four candles. For this linear lateral case, the four candles were identified in the image, obtaining an image that shows the faces exposed to the radar system.

Figure 9 displays the reconstructed image for the circular geometry using a single candle with a diameter of 4 cm and another measurement of two candles close together. For the two circular measurement geometry cases shown in Figure 9, it was possible to identify the candle contour through the image provided by the radar at 100 GHz. The quasi-monostatic geometry improved the image contour accuracy. The image of the inside part of the candle was rough because the penetrability of wax at mmW frequencies is around several wavelengths, giving a penetrability of 2 mm at 100 GHz. The image resolution was consistent with the wavelength used in the measurements, as could be observed here.

Comparing the results obtained for the linear lateral view and the circular frontal view, the objects’ contours were detected with a higher level of accuracy for the circular frontal measurement scenario because the target was observed from a different angle of view, thereby obtaining more information. 

## 5. Conclusions

This paper presented a linear and circular UWB millimeter-wave and terahertz monostatic near-field synthetic aperture imaging system for the exploration of optically opaque non-metallic targets [12,18]. The developed and applied system was shown to be capable of providing images using linear and circular measurement geometries that match the frontal and lateral views in vehicles. 

Subsequently, this paper proposed and proved the robustness of the UWB millimeter-wave and terahertz monostatic near-field synthetic aperture imaging system in the frequency domain [8,9,10,11,12] (Equation (4)) as an imaging technique, using 35 GHz and 38% absolute and fractional bandwidths at 100 GHz, as well as 110 GHz, and 40% absolute and fractional bandwidths at 300 GHz. The proposed expression (Equation (4)) takes into account the impact of the frequency ($f^{2}$ is introduced) on the coherent imaging reconstruction, which proves both its accuracy and its capability to deal with realistic surfaces (with a roughness above λ/10). While the introduction of the frequency dependence $k_{b}^{2}(f)$ in the denominator of Equation (4) may be proven from a wrapping geometry [10], its effect on more limited scanning geometries was not properly studied previously in detail with the approach presented in this paper. Thus, this paper develops the possibility to have two separated broadband interrogating frequency bands (100 and 300 GHz) with the aim of providing a unique capability to test for its effect.

To the authors’ best knowledge, there are no previous experimental results that proved a high relative frequency-dependent reconstruction at these frequency ranges with real surface roughness objects using low-contrast materials. These materials are non-metallic low-relative-permittivity-environment objects that match the realistic approach in vehicular scenarios.

For the linear geometry, the technique was tested to obtain internal images of penetrable geometries (Figure 6 and Figure 7) at 100 GHz (75–110 GHz) and at 300 GHz (220–330 GHz). The results show that the radar is capable of rebuilding the surfaces exposed to the radar sweep. The best images were obtained at the 300 GHz band due to its shorter wavelength and the bandwidth (frequency sweep). 

The linear and circular measurement scenario results for different sizes of candles, representing different urban objects, provide confidence regarding the feasibility of using this system and techniques in sensing applications operating at the 100 GHz band and the 300 GHz band, depending on the final application.

## Figures and Tables

**Table 1:** The 100 GHz parameters. IF—intermediate frequency.

Parameter	Value
Initial frequency	75 GHz
Final frequency	110 GHz
Number of sampling points	8192
IF bandwidth	1 kHz
Output power	0 dBm

**Table 2:** The 300 GHz parameters.

Parameter	Value
Initial frequency	220 GHz
Final frequency	330 GHz
Number of sampling points	8192
IF bandwidth	30 Hz
Output power	0 dBm

**Table 3:** Measurement configuration for linear geometry.

	Distance d (cm) in *x*-Axis Positions in *x*-Axis 100 GHz	Distance d (cm) in *x*-Axis Positions in *x*-Axis 300 GHz	Size (cm) Height × Width
Post-it	21.1	24.5	7.8 × 7.8
Green cube	21.1	24.5	7 × 7
Box (empty)	240	501	6.5 × 8.8
Box with ball	240	501	6.5 × 8.8
